# Comparison of the effects of two laser photobiomodulation techniques on bio-physical properties of *Zea mays* L. seeds

**DOI:** 10.7717/peerj.10614

**Published:** 2021-01-15

**Authors:** Mohammed Hasan, Marlia M. Hanafiah, Intsar H.H. Alhilfy, Ziad Aeyad Taha

**Affiliations:** 1 Department of Earth Sciences and Environment, Faculty of Science and Technology, Universiti Kebangsaan Malaysia, Bangi, Selangor, Malaysia; 2 Centre for Tropical Climate Change System, Institute of Climate Change, Universiti Kebangsaan Malaysia, Bangi, Selangor, Malaysia; 3 Department of Field Crop Science, College of Agriculture, University of Baghdad, Baghdad, Iraq; 4 Institute of Laser for Postgraduate Studies, University of Baghdad, Baghdad, Iraq

**Keywords:** Maize, Field performance, Seed quality, Nd:YAG laser, He–Ne laser

## Abstract

**Background:**

Laser applications in agriculture have recently gained much interest due to improved plant characteristics following laser treatment before the sowing of seeds. In this study, maize seeds were exposed to different levels of laser treatment prior to sowing to improve their field performance. The aim of this study is to evaluate the impact of pre-sowing laser photobiomodulation on the field emergence and growth of treated maize seeds.

**Methods:**

The maize seeds were first photobiomodulated with two lasers: 1) a helium-neon (He–Ne) red laser (632.8 nm), and 2) a neodymium-doped yttrium aluminum garnet (Nd:YAG) green laser (532 nm). Following three replications of randomized complete block design (RCBD), four irradiation treatments were applied (45 s, 65 s, 85 s, and 105 s) at two power intensities (2 mW/cm^2^ and 4 mW/cm^2^).

**Results:**

Based on the results, maize seeds pretreated with a green laser and 2 mW/cm^2^ power intensity for 105 s exhibited the highest rate of seed emergence (96%) compared to the untreated control seeds with a lower seed emergence rate (62.5%). Furthermore, maize seeds treated with a red laser for 45 s showed an increased vigor index compared to the other treatment options and the control (*P* < 0.01). The treatment groups also showed statistically significant differences in seedling growth characteristics compared to the control group *p* < 0.01. The green laser produced a significant enhancement of about 24.20 cm in seedling length, 8.2 leaves/plant, and 3.4 cm in stem diameter compared to the untreated seeds. Moreover, the green laser treatment showed 57.4 days to anthesis, which was earlier than the untreated seeds (61.4 days). The results showed that the protein, oil, and starch contents of the seeds irradiated with the green laser were 17.54%, 6.18%, and 73.32%, respectively, compared to the seeds irradiated by the red laser with 16.51%, 6.33%, and 71.05%, respectively.

**Conclusions:**

The photo biomodulation of maize seeds using a green laser light can improve the field emergence, seedling growth, and seed quality of the treated seed compared to the red laser treatment.

## Introduction

Since its first demonstration in 1960, the laser has been used in numerous scientific, military, and industrial applications (Hernandez-Aguilar, Dominguez-Pacheco & Cruz-Orea, 2015). Recently, the laser was used in agriculture for photobiomodulation because its application has less impact on the course of genetically-controlled biological processes (He et al., 2017). When applied at low intensity, laser light exerts a photobiomodulation effect on seedlings (Chen et al., 2005). In any plant physiological stage, this photo biomodulation mechanism relies on the synergy between the photoreceptors and the polarized monochromatic laser beams ( Hernandez-Aguilar et al., 2010; Perveen et al., 2010). Studies have also demonstrated the photo biomodulation effect of laser radiation on various plant tissues and organs (Vasilevski, 2003).

Maize (*Zea mays* L.) is one of the basic crops in which seed quality determines the level of seedling emergence and crop establishment (Hamood & Elsahookie, 2011; Zowain, 2014). Obtaining a high crop yield requires high-quality seeds that can produce rapid and uniform seedling emergence (Hernandez-Aguilar, Dominguez-Pacheco & Cruz-Orea, 2015). Seed vigor is the physiological quality that related to rapid, uniform seedling emergence, and development under different soil conditions (Hasan et al., 2020a; Hasan et al., 2020b).

Because the production of low maize seed vigor is a significant problem in the seed industry, it is essential to develop a method or technique that can be applied as a pre-sowing treatment to improve seed vigor and its field performance. Laser irradiation treatment of seeds is one method for enhancing crop yield (Hernandez-Aguilar, Dominguez-Pacheco & Cruz-Orea, 2015). However, the experimental designs under current use have employed only two readily available laser wavelengths (i.e., 632.8 nm and 532 nm) for the irradiation process. Based on an extensive review of the existing literature, this study is the first on the effects of laser irradiation at those two wavelengths on the characteristics of *Zea mays* L. Thus, this study aims to examine the field emergence, growth, flowering, and seed quality parameters of laser beam-irradiated maize seedlings and compare the effects of two types of laser photobiomodulation on the biophysical properties of *Zea mays* L seeds. The treatment was conducted for various irradiation period (45 s, 65 s, 85 s, and 105 s) at two power intensities of 2 mW/cm^2^ and4 mW/cm^2^.

### Previous studies related to seed performance induced by laser photobiomodulation

Different types of lasers have been studied for their effects on seed germination and growth of plants. The results showed that the treatment improved the sowing qualities of the seeds, produced more vigorous plants, reduced the number of plant development phases, increased both seed and stem yields to a significant level, and improved the rate of germination by 10–15% (Hernandez-Aguilar, Dominguez-Pacheco & Cruz-Orea, 2015; Hoseini et al., 2013; Jia & Duan, 2013; Pérez Reyes et al., 2015). Another study focused on the stimulating effect of radiation on plant height, stalk thickness, height at first ear formation, number of leaves, and leaf area (Podlesna et al., 2015; Srećković et al., 2014; Tang et al., 2019). Higher mutation frequency has also been reported in plants derived from wet seeds compared to plants from irradiated dry seeds (Jamil et al., 2013; Levskaya et al., 2009).

Seed germination can also be affected by exposure to laser irradiation before sowing. Among the advantages of laser treatment include the low risk of organ damage and the control of disease. (Álvarez et al., 2011; Ferdosizadeh et al., 2013; Soliman & Harith, 2009). As a physical process, laser stimulation involves the ability of plants to absorb and store radiant energy. The seeds first absorb the light energy before transforming it into chemical energy for growth (Joshi, Joshi & Agrawal, 2012). Generally, lasers are devices that emit light with specific optical properties such as intensity, wavelength, and divergence. Scientists are still baffled by the anticipated benefits of laser irradiation (Aguilar et al., 2015; Hernandez-Aguilar, Dominguez-Pacheco & Cruz-Orea, 2015; Jakubiak & Gdowska, 2013; Metwally et al., 2013; Muszyñski & Gladyszewska, 2008).

Hence, more studies have focused on the physical factors that could be used in preparing seed lots (Khalifa & El Ghandoor, 2011; Mohssen, Sharafi & Siadat, 2010; Rimal et al., 2014; Taie et al., 2014). To this aim, laser light has been particularly useful, owing to its specific characteristics (Hernandez-Aguilar et al., 2009). Existing studies suggested a positive influence of pre-sowing seed exposure to laser beams on the induction of seed germination, initial seed development, and yield of selected seeds (Chen et al., 2005; Ćwintal, Dziwulska-Hunek & Wilczek, 2010; Hasan et al., 2020a; Li et al., 2013; Podlesna et al., 2015). However, little is known about the mechanism through which laser beams influence seeds and plants because few studies address it (Álvarez et al., 2011; Hernandez-Aguilar, Dominguez-Pacheco & Cruz-Orea, 2015; Podlesna et al., 2015). Therefore, studies are needed on the biochemical and physiological processes involved in laser-irradiated seeds and the resulting plants.

In nature, plants often experience harsh environmental conditions, such as drought, salinity, and chilling. These conditions can delay growth and development, reduce yield, and in extreme cases, kill the plant (Hasan, 2018; Hasan et al., 2020b). Environmental conditions are significant factors that must be controlled during the life cycle of the plant, and various tolerance mechanisms have been suggested based on the biochemical and physiological changes (Fadillah & Marlia, 2016; Hanafiah et al., 2019; Harun & Hanafiah, 2018; Muhammad-Muaz & Marlia, 2014).

## Materials & Methods

### Research location and planting materials

This study was conducted at the experimental field located at the Faculty of Agriculture and Laser Laboratory, Institute of Laser for Postgraduate Studies, University of Baghdad (33°16′26″N, 44°22′39″E). The Office of Agriculture Research provided the seeds used for the study (Baghdad 3 cultivar).

### Weather conditions and soil properties

The experimental site has a hot, dry climate and an average annual (2018) temperature of 34 °C. The soil characteristics at the experimental field were: pH of 7.2, Ammonia nitrogen of 0.009%, total Nitrate of 0.0017%, total K of 1.6 mg/l, available P of 43.2 mg/kg, soil electrical conductivity (EC) of 3.8 meq/100g, silt of 28.9 g/kg, clay of 28.5 g/kg, sand of 32.6 g/kg, and a texture of silty clay.

### Laser setup and treatments

Seeds were stimulated with the He–Ne (red) laser and Nd:YAG second harmonic generation (green) laser. Power densities were 5 mW/cm^2^ and 300 mW/cm^2^, respectively. The laser beams were focused from the side onto the seeds and equally distributed underneath. The exposure time was measured with a timer. Optics in the lasers were used to ensure optimum laser power. The laser’s irradiation power intensity was 2 mW/cm^2^ and 4 mW/cm^2^. The intensity was measured using a power meter. The laser was used to irradiate the maize seeds for exposure times of 45, 65, 85, and 105 s.

### Experimental design

The experiment consisted of (1) a randomized complete block design (RCBD), (2) two types of lasers (the Nd:YAG second harmonic generation (green) and the He–Ne (red)), (3) four irradiation times (T_1_ = 45, T_2_ = 65, T_3_ = 85, and T_4_ = 105 s), (4) two laser intensities (S_1_ = 4 mW/cm^2^ and S_2_ = 2 mW/cm^2^), and (5) three replicates. Row to row spacing was 75 cm. Maize plants were sown with a spacing distance of 25 cm within the plants. A total of 1,200 seeds were irradiated. The sowing of seeds occurred in the last week of July, while harvesting was conducted in the first week of November. Basal fertilizers were applied during sowing at the following rates: diammonium phosphate (DAP) (46:18 of N: P_2_O_5_) at 436 kg/ha, and urea at 520 kg/ha (half his rate 10 days after sowing, and the rest at the flowering stage). Weeding was done manually during the growing period, while insects were controlled chemically using diazinon pesticide at the rate of 6 kg/ha and applied at 20 and 35 days after sowing. The drying of the ears was done naturally in a shaded, ventilated place. The ears were manually de-grained when the grain’s moisture content was approximately 15% and stored at a temperature of 25 °C. The seed lots were first standardized by size and color. A sodium hypochlorite solution (1% v/v) was used for sterilization before irradiation. From each treatment group, six seeds were selected and measured for thicknesses using a vernier caliper.

### Plant parameters

Several parameters were estimated after pre-sowing seed stimulation with laser light. After all the viable seeds had emerged (no new seed emergence was observed), a daily count of seedling emergence was conducted while the other seedling parameters (emergence %, emergence index, and vigor index) were recorded. The mean emergence time (MET) was calculated as follows: [MET = Σ*Dn*∕Σ*n*] where D = number of days from the first day of sowing, and *n* = the number of emerged seeds per day. The emergence index (EI) was calculated using the formula provided by the Association of Official Seed Analysis as follows: “[EI = No. of emerged seeds/Day of first count +⋯ + No. of emerged seeds/Day of final count]. The emergence speed was calculated by: [Es = Σ Ni/Di], where Ni = number of seeds emerged per day, and Di = Number of days (daily germination). The seedling vigor index was calculated using the following formula: Vigor Index (VI) = emergence (%) × Seedling length (cm).

Seedling length is a measure of the distance from the soil’s surface to the flag leaf-bearing nodes (21 days after sowing). The top ear was determined from five randomly selected plants in a plot, and the mean of the measurements was determined and presented as the seedling length. The flowering date is the date on which 100% of the plants in a plot attained anthesis. Incipient silk extrusion was recorded and expressed as days after planting. The stem diameter and number of seedling leaves were also measured. The proximate composition of the grain, including protein, starch, and oil, was analyzed after harvesting based on the method prescribed in  American Association of Cereal Chemists (2000).

### Statistical analysis

The data acquired in the study were analyzed using Genstat® Statistical software version 19. The software was used for the analysis of variance (ANOVA) at a significance level of *p* < 0.01. Fisher’s test was used to define the observed levels of significant differences between the dataset means. All measurements were performed in triplicate to reduce errors.

## Results

### Field performance

**Figure 1:**
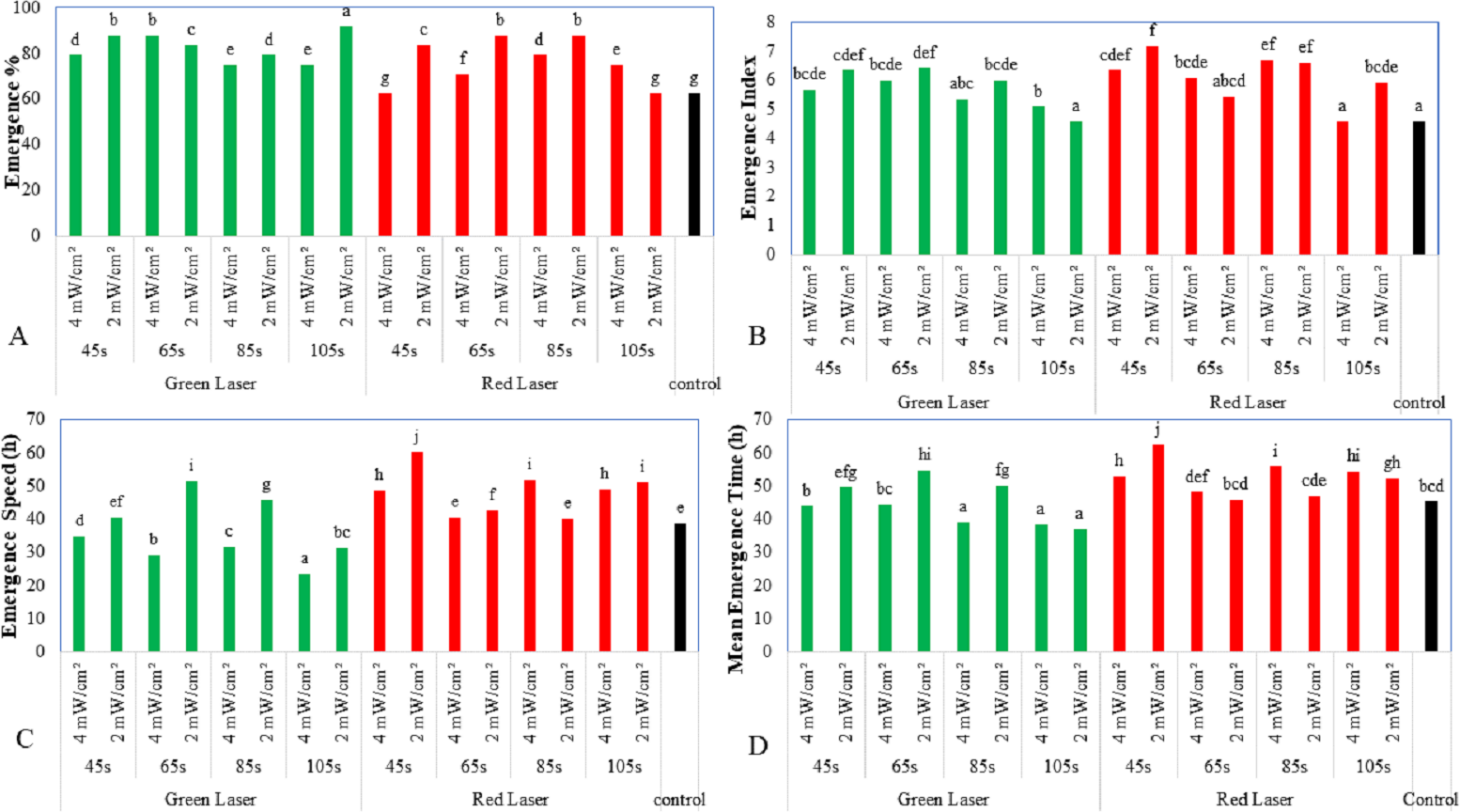
Effect of two wavelengths of laser irradiation at different exposure times and laser intensity on field performance parameters. (A) Emergence %, (B) emergence index, (C) emergence speed, and (D) mean emergence time. Means of treatments were compared using the least significant difference (LSD) at *p* ≤ 0.01. Mean values with the same letters are statistically equal (Fisher’s, *α* = 0.01).

The effects of the maize laser treatments on its field performance parameters are shown in Fig. 1. The results showed that laser treatment at different exposure times and power densities significantly affected the emergence % of maize seeds. The emergence percentage of the treated seeds improved, compared to the untreated seeds. Pre-sowing irradiation with the green laser at 2 mW/cm^2^ for 105 s brought about significant improvement in the emergence rate (91.67%) compared to the untreated seedlings (62.5%) (Fig. 1A). The red laser treatment also had similar effects, with the highest emergence rate of 87.5% achieved with red laser irradiation (2 mW/cm^2^) for 85 s.

Figure 1B shows the improvement in the emergence index of maize seeds following laser treatments. Laser treatment at the two studied intensities improved the emergence index of the seeds. The best performance (7.17%) was observed with the red laser for 45 s exposure and power of 2 mW/cm^2^. Next was the seeds irradiated by the red laser for 85 s at 4 mW/cm^2^ (6.67%). The untreated seeds recorded the lowest emergence index (4.58%). Figure 1C shows the effects on the emergence speed of using different laser types and exposure times. Exposure to the green laser (4 mW/cm^2^) for 105 s brought a significant reduction in emergence speed (23.44 h) compared to the unexposed seeds (38.6 h).

**Figure 2:**
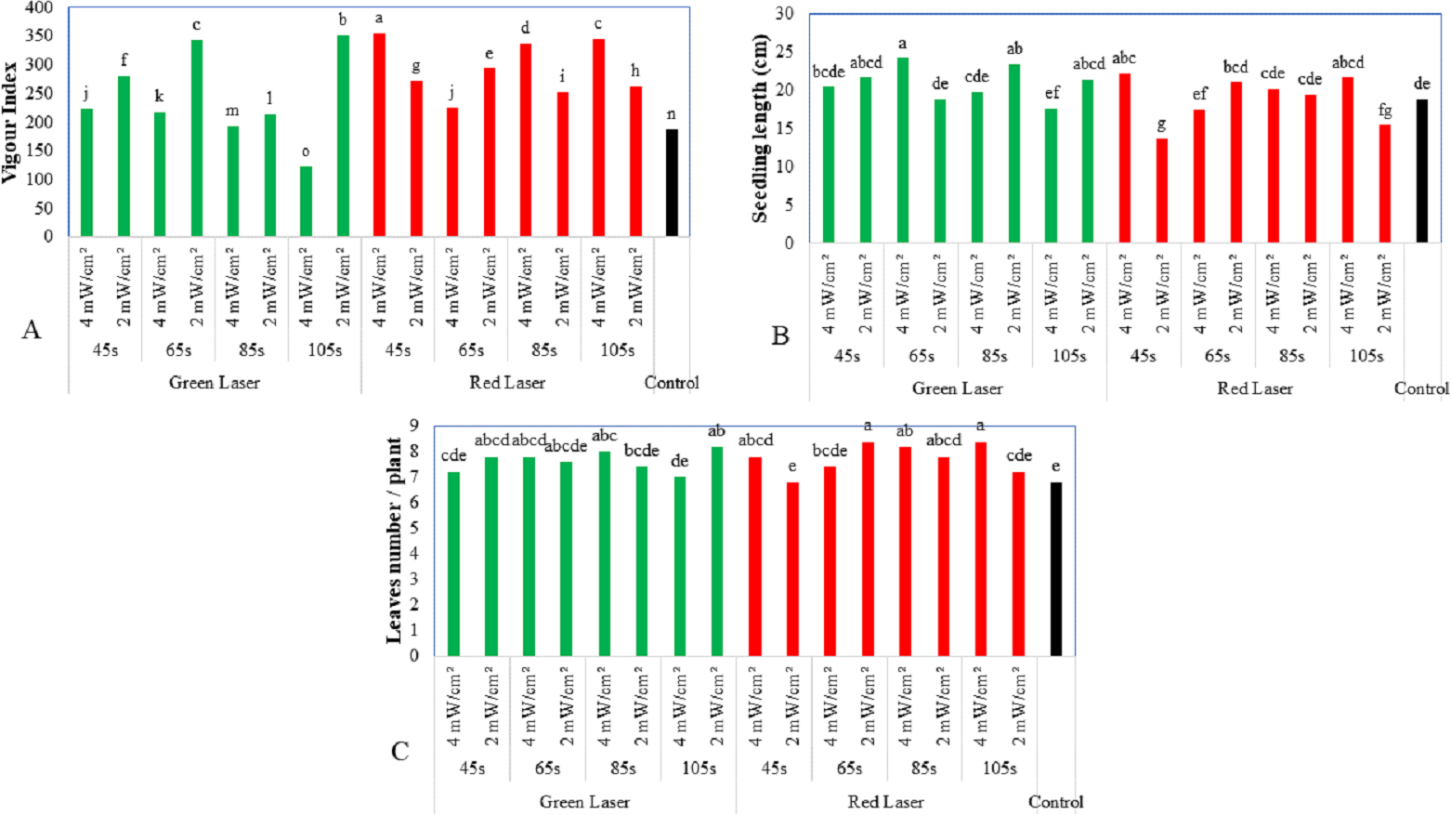
Effect of two wavelengths of laser irradiation at different exposure times and laser intensity on the maize seedling performance. (A) Vigour index, (B) seedling length, and (C) leaves number parameters of maize seedling. Means of treatments were compared using the least significant difference (LSD) at *p* ≤ 0.01. Mean values with the same letters are statistically equal (Fisher’s, *α* = 0.01).

The exposure of maize seeds for 105 s to the green laser (2 mW/cm^2^) significantly reduced the mean emergence time (37 h) compared to the exposure time for the red laser (52.24 h). There was no significant difference compared to the control at 45.33 h (Fig. 1D). As shown in Fig. 2A, the vigor index of the treated maize seeds exhibited substantial differences. Hence, laser treatment at different wavelengths and exposure times improved the vigor index of the plants. The highest vigor was exhibited by seeds exposed to 4 mW/cm^2^ of red laser intensity for 45 s (355.2), followed by seeds treated with 2 mW/cm^2^ of green laser intensity (351). The untreated seeds showed the lowest vigor (188).

### Growth parameters

Figure 2B shows the results of green laser treatment for 65 s. The green laser promoted the maximum maize seedling length (24.2 cm), while the red laser treatment for 45 s yielded a minimum height of 13.6 cm. All the laser treatments for different exposure times (45, 65, 58, and 105 s) increased the seedling length of the maize plant when compared to plants from the untreated seeds (18.8 cm). Seeds treated with the green laser for 45 s recorded the largest stem diameter, even though there was a significant difference compared to the control. The highest number of leaves per plant (8.4 leaves/plant) was observed in plants from seeds treated with the red laser for 65 and 105 s, followed by plants from seeds treated with the green laser for 105 s and the red laser for 85 s (8.2 leaves/plant) compared to the plant from the untreated seeds (6.8 leaves/plant) (Fig. 2C).

**Figure 3:**
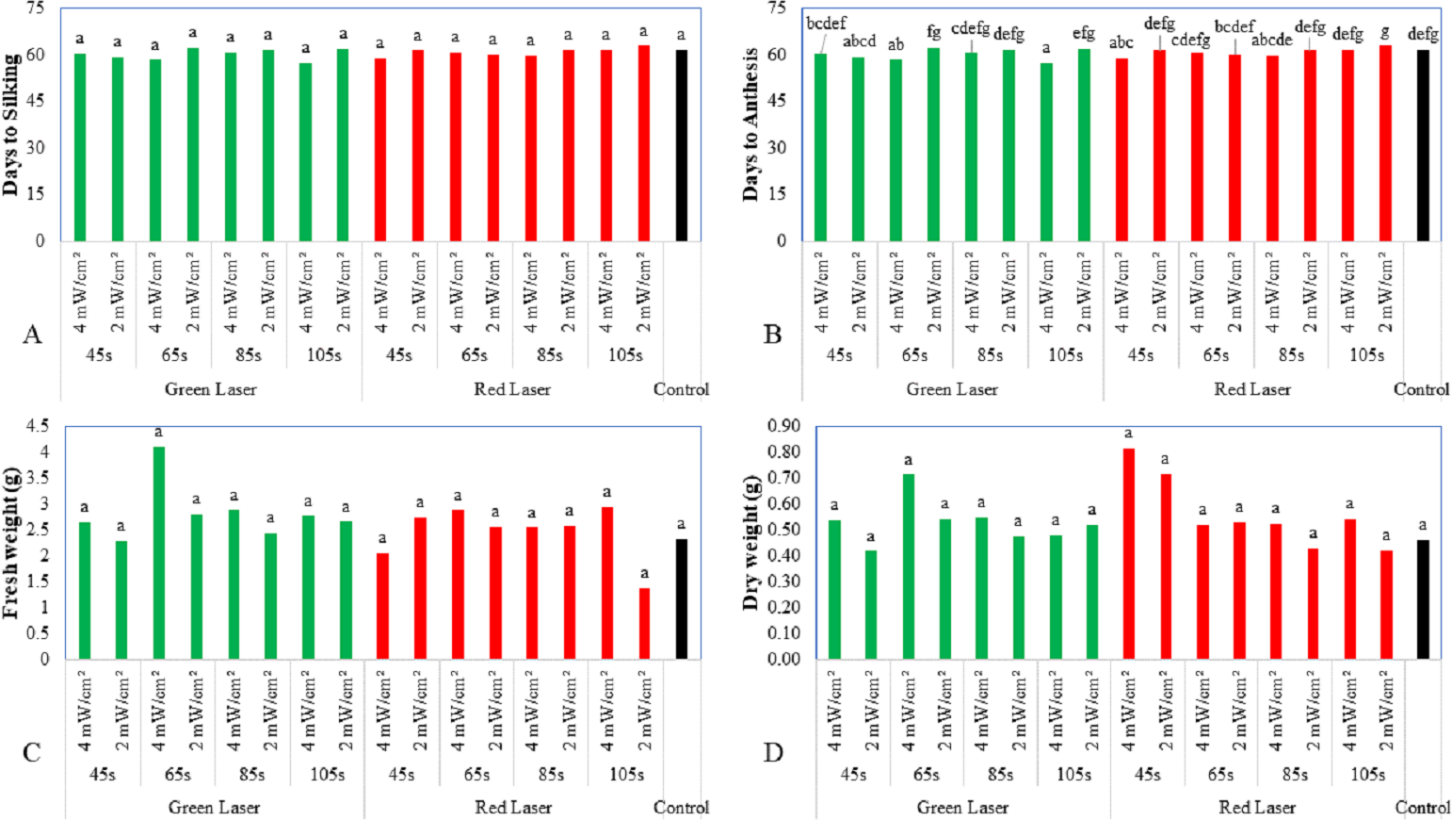
Effect of two wavelengths of laser irradiation at different exposure times and laser intensity on the flowering parameters as well as fresh and dry weight parameters. (A) Days to silking, (B) days to anthesis, (C) fresh weight, and (D) dry weight parameters of maize seedling. Means of treatments were compared using the least significant difference (LSD) at *p* ≤ 0.01. Mean values with the same letters are statistically equal (Fisher’s, *α* = 0.01).

Not all laser radiation treatments showed a significant effect on the silking of maize plants compared to the control (Fig. 3A). The seeds treated with the red laser for 105 s showed a delay in the time to silking (62.8 days), followed by seeds treated with the green laser for 65 s (62.3 days), and compared to the control (61.4 days). The shortest period to silking was observed in the seeds irradiated with the green laser for 105 s (57.4 days). Seeds of various types and durations of laser radiations resulted in fewer days to anthesis. The shortest durations (57.4 and 58.3 days) were observed with 105 s of treatment at 4 mW/cm^2^ of the red laser and 65 s at 4 mW/cm^2^ of the green laser. The control showed 61.4 days to anthesis (Fig. 3B).

Figures 3C and 3D show the effects of laser radiation on fresh and dry maize seedlings. Based on the ANOVA, there were no significant differences between the various treatments. The maximum fresh weight (4.1 g) was observed with the green laser radiation for 65 s at 4 mW/cm^2^ compared to the control (2.3 g). The best results for the dry weight were obtained with the red laser for the short exposure time of 45 s (0.81 g) and the green laser for 65 s (0.71 g). By comparison, the control was 0.46 g.

### Seed quality

**Figure 4:**
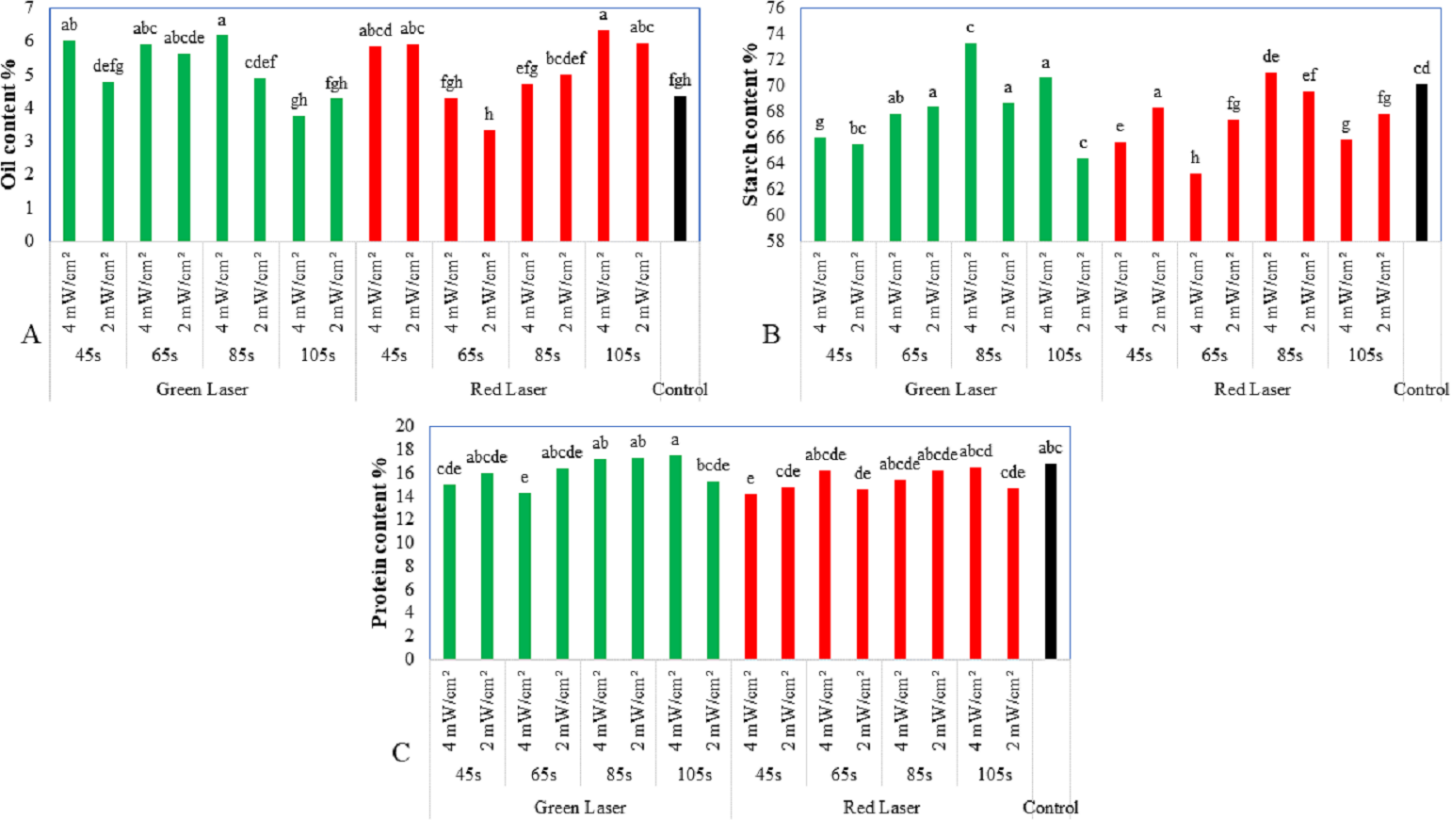
Effect of two wavelengths of laser irradiation at different exposure times and laser intensity on the maize seeds quality. (A) Oil content, (B) starch content, and (C) protein content of maize seeds. Means of treatments were compared using the least significant difference (LSD) at *p* ≤ 0.01. Mean values with the same letters are statistically equal (Fisher’s, *α* = 0.01).

Figure 4 shows the effect of laser exposure on the oil content of seeds. The laser-treated seeds and the control seeds presented a comparable level of oil content. As shown in Fig. 4A, there were significant differences in the oil content of the seed. Generally, the oil content of seeds increased with exposure to the laser for different exposure times. Seeds exposed to a red laser for 105 s at 4 mW/cm^2^ l intensity showed higher oil content (6.33%), followed by those exposed to the green laser for 85 s at 4 mW/cm^2^ intensity (6.18%), and the control seeds (4.37%). The highest levels of total protein and starch content were recorded in seeds exposed to the green laser compared to the control (*p* < 0.01; Fig. 4B). The exposure of maize seeds to a laser at different exposure times had a significant influence on the protein content of the seeds (Fig. 4C). An increase in the green laser’s exposure time was generally found to increase the protein content of the seeds, with the highest protein content of 17.52% observed in seeds exposed to 4 mW/cm^2^ laser power for 105 s. Next was 17.35% for seeds exposed to 2 mW/cm^2^ laser power for 85 s. The control seeds had a protein content of 16.8%. The protein content increased gradually, whereas the starch content showed maximum activity at 85 s. These results were found to be significant (*p* < 0.01) compared to the control group.

## Discussion

### Field performance

This study examined the effect of pre-sowing laser stimulation on the emergence of the maize seeds of varying initial quality. The laser light treatment increased the emergence percentage and index. The results of the green laser light stimulation showed an increase in the emergence percentage. The highest observed increase in this parameter depended on the time of exposure. The vigor index, or the capacity of seeds to emerge quickly, has a practical aspect. A higher vigor index often manifests in the more robust development of the seedlings, increased fresh weight of the whole seedling, and after that, the plant.

Phytochromes are involved in the sensing of light by the seeds. The control of germination using red and far-red light was one of the earliest phytochrome-mediated responses that could affect the embryo’s growth capacity or the constraint imposed by seed tissues around it. There is substantial evidence on the role of phytochromes in promoting the synthesis of gibberellins (GAs), a critical stimulant for germination (García-Martinez & Gil, 2001; Hilhorst, 2018; Ribalta et al., 2019). Phytochromes also play a role in regulating the sensitivity to GAs (Mishra & Khurana, 2017). Recently, phytochromes have also been shown to affect the degradation of abscisic acid, the critical plant hormone that maintains dormancy (Miransari & Smith, 2014).

It is generally accepted that the germination process is sensitive to irradiation of various wavelengths of visible and infrared light. For example, red light could act on the phytochrome system (photoreceptor), promoting germination (Shichijo et al., 2001). Both the breaking of dormancy and the stimulation of germination with laser treatments have focused on several cereal grains and vegetable seeds. The experimental evidence suggests significant positive effects that improve the quality of plant products obtained from these irradiated seeds.

Laser pre-sowing treatment of seeds has recently received attention (Hernandez et al., 2010). Studies have already confirmed the positive influence of laser treatment on plant characteristics (Hoseini et al., 2013; Vasilevski, 2003). The laser energy is absorbed by the exposed seeds during laser treatment and further transformed into chemical energy for subsequent utilization during germination and growth (Aladjadjiyan, 2012). It is believed that the positive impact of laser pre-sowing seed treatment is due to the energy supplement during laser treatment (Hernández & Michtchenko, 2011). Given that non-photosynthesizing cells can also accumulate light energy (Hasan et al., 2020a; Hasan et al., 2020b), they may affect the biomolecules at the germination stage, leading to decreases in the germination parameters (Ambika, 2007). Because plants react positively to the light irradiation at wavelengths of 630 nm–650 nm, it is possible to use laser light irradiation for the pre-sowing treatment of seeds to improve the germination capacity and strengthen the vigor of young plants or seedlings in the early stages of development (Hernandez-Aguilar et al., 2011).

Previous studies show that, in some cases, the mean emergence time of a single seed subjected to laser irradiation can be accelerated by more than 37 h compared to the control. Therefore, the period of plant growth from irradiated seeds can be longer compared to the control. This longer period can further impact seed quality. The increase in emergence further increases as laser exposure time increases. Similar changes in seed emergence during germination due to pre-sowing laser treatment have already been reported in wheat and maize (Srećković et al., 2014). The same authors reported that the increase in germination could be due to increased internal seed energy from laser treatment stored in metabolic compounds.

The results obtained in this study indicate that pre-sowing laser light stimulation affected the increase and acceleration of the emergence of seedlings obtained during varying exposure times of maize seeds. Similar effects were observed by Hernandez-Aguilar et al. (2009) due to the irradiation of the maize seeds with low initial germination capacity.

On the other hand, Hernandez-Aguilar et al. (2009) conducted experiments using the He–Ne laser irradiation at a power density of 20 mW/cm^2^. An exposure time of 60 s resulted in increased maize seedling emergence. It also increased the mean for the time of emergence compared to the exposure times of 30, 120, 180, 300, and 600 s. Pérez Reyes et al. (2015) showed that pre-sowing treatment of spring barley seeds with the laser light at a power density of 1 mW/cm^2^ and an exposure time of 30 min improved the quality and quantity of the grain.

Studies by Ambika (2007) and Asghar et al. (2016) on pre-sowing laser treatment also reported similar effects of a 20–35% increase in the rate of seed germination, possibly due to changes in the physiological state of the seeds and plants on exposure to different intensities of laser light. These changes in plant physiology can either stimulate or inhibit their development, depending on the power of the laser light (AlSalhi et al., 2018). Other factors that could contribute to the improvement in seed germination is the energy transfer processes involved seed development (Qiu et al., 2013). Laser radiation has also been studied for its spectral effect on seed germination by Chen et al. (2005). The study reported that laser light-induced changes in the plant functions and activated faster cell division, thereby resulting in an initially faster rate of seed growth and development.

The germination process is known to be sensitive to different light wavelengths. For example, blue and red lasers have been known to act on the phytochrome system (photoreceptor) to promote seed germination (Dănăilă-Guidea et al., 2011). The ability of laser radiation to break the dormancy phase of seeds and induce germination has been reported for several cereals and vegetable seeds. The experimental findings suggest a significant improvement in the quality of plant products produced from irradiated seeds. The studied species demonstrated the positive effect of the He–Ne laser irradiation on seed germination and development (seed height) compared to the control.

### Growth parameters

Generally, the exposure time of the maize seeds to laser light for 45 s, and 105 s had significant effects on the analyzed parameters. On the other hand, in some indices (seedling length, number of leaves, stem diameter, and fresh and dry seedling weight), the most beneficial values were obtained from seeds irradiated by the green laser. The results of other studies showed that the combination of exposure time to the laser light and the surface power density might have a significant effect on seedling growth parameters; the longer the exposure time at a higher dose, the more beneficial the effect (Muthusamy et al., 2012).

Figures 3A to 3D show the impact of green laser irradiation of maize seedlings on seedling length. The results showed that green laser irradiation of maize seedlings improved seedling length. However, seeds irradiated with the red laser had more leaves per plant than the control. Seeds irradiated with the green laser showed smaller stem diameters than the seeds irradiated with the red laser and the control seeds. Seeds irradiated with the green laser also showed shorter anthesis periods than seeds irradiated with the red laser and the control seeds.

Salyaev et al. (2007) and Hernandez et al. (2010) concluded that two specific responses could be induced when cells are exposed to laser light: (a) a rapid stress effect that increases the level of lipid peroxidation products generated, and (b) a series of secondary reactions resulting from adaptive metabolic changes that can elicit some morphogenetic processes. For example, according to Chen et al. (2005), the He–Ne laser pretreatment can improve the inner energy of seeds, lead to an enhancement of cotyledon enzymes, and accelerate the cell’s metabolism. These factors significantly increased the cycles of cell division (mitosis), which resulted in an increase in the length of the plant organs during early growth.

Few studies on the influence of laser radiation on plant materials have demonstrated the effect of laser light on plant characteristics (Abu-Elsaoud & Tuleukhanov, 2013; Hernández & Michtchenko, 2011; Muthusamy et al., 2012). Most studies have focused on the influence of the He–Ne gas laser irradiation. Szajsner & Drozd (2007) compared the effects of laser light on the early stages of Spring wheat (Banti variety) development. Semiconductor laser light provided more efficient grain irradiation, which, in turn, led to a better germination rate and induction of the morphological features of the resulting seedlings.

Improved cell metabolism supports the germination process. This process occurs when seeds are irradiated with natural light, such as sunlight, since it also contains the red light, but it is incomparably much more pronounced in the case of seed irradiation by coherent, polarized red light such as laser light (Asghar et al., 2016). Apart from the rapid absorption of red light, the effects of laser beams also result in the transfer of energy to the seed that accelerates metabolism and supports the germination process (Metwally et al., 2013). That is why lasers, not only red but other wavelengths, improve germination and growth (Nasim & Jamil, 2014). Another reason for the positive effects of laser pre-treated seeds is the reduction of free radical concentrations. Reduced free radicals improve resistance to hostile environments such as drought, chill, and humidity (Śliwka, 2014).

The seeds’ response to laser irradiation depends on several factors, including laser wavelength, duration of a single exposure, repetition of the irradiation, laser intensity, the optical properties of seeds, and plant variety. Although the reaction to a red laser is typically more intensive than the reaction to other wavelengths, the optimum regime of irradiation is still to be found for each plant variety. The influence of pre-sowing laser irradiation on germination and plant growth is thoroughly documented in the case of grains (Muszyñski & Gladyszewska, 2008; Perveen et al., 2010). Enhanced germination was also reported in the case of some vegetables (Kouchebagh et al., 2014b; Osman, El-Tobgy & El-Sherbini, 2009).

The effect of various lasers on plant growth has recently received increased attention and has elicited some preliminary studies (Joshi, Joshi & Agrawal, 2012; Samuilov & Garifullina, 2007; Tang et al., 2019). Notably, most previous studies did not consider the analysis of the positive restorative effects of He–Ne laser treatment on plant photosynthesis. In the visible range, low laser doses appear as small amounts of heat. The photobiomodulation effects of the He–Ne laser could be due to electromagnetism (Álvarez et al., 2011; Chen et al., 2005; Jamil et al., 2013) because the treatment primarily involves energy absorption at the molecular level, affecting plant physiology. The photobiomodulation effect of laser beams is documented as a function of the applied wavelength, irradiation dose, and irradiation time (Álvarez et al., 2011). The He–Ne laser typically provides better performance due to the higher sensitivity of the photoreceptor phytochromes in plants to red light (Tang et al., 2019).

Figures 2A to 2C and 3A to 3D show that the growth of seedlings from laser-irradiated seeds at 530 nm was statistically different from that of the untreated plants. As reported by AlSalhi et al. (2018), laser treatment improves plant length and the dry weight of wheat seedlings. The outcome of the present study agreed with the earlier report by Podlesna et al. (2015) on the impact of laser irradiation on the growth and flowering of the faba bean and white lupine. The treated seeds were observed to grow more quickly in mass during imbibition than to the control samples.

Similarly, the dry weight, seed emergence rate, flowering time, and time to maturity have been reported to increase laser pre-sowing treated seeds of various leguminous species (Asghar et al., 2016). Other studies have reported positive effects of seed pre-treatment with laser radiation before sowing on growth parameters (Álvarez et al., 2011; Jia & Duan, 2013; Khalifa & El Ghandoor, 2011). The stimulatory effect of laser treatment is attributed to the phytochrome mediated non-photosynthetic transformation of light energy. Phytochrome is a component of a photoreceptor system in plant cells that is localized at the plasmalemma, mitochondrial, chloroplast membranes, and endoplasmic reticulum (Sanchez-Hernandez et al., 2015). Phytochrome is involved in the regulation of oxidative and photosynthetic phosphorylation (Michtchenko & Hernandez, 2010). Phytochrome is also involved in energy-transduction to the plasmalemma, tonoplast, and endoplasmic reticulum. This process is where light-induced membrane potential is generated, and light energy is transformed into the transmembrane. This transformation implies that, in addition to the photoreceptors responsible for light absorption, there is also a system of inter-linked membrane structures operating within the cell that accounts for the transformation and utilization of the absorbed light energy (Hwida, Metwally & Lobna, 2012).

### Seeds quality

Different techniques have been developed for the enhancement of maize seed germination in poor soil conditions. This study was conducted to investigate the effect of laser irradiation on various characteristics of the maize plant. The results of the pre-sowing treatment revealed positive effects on seed quality (Fig. 4).

Figure 4 shows that the maize seeds’ exposure to the green laser improved the protein content of the seeds, compared to the seeds irradiated by the red laser and the control seeds. The seeds’ starch content was also affected by the green laser; green laser-irradiated seeds had more starch content compared to the red laser-irradiated seeds and the control seeds. The effect of the red laser treatment was more significant on the oil content of the maize seeds compared to the seeds treated with the green laser and the control seeds (Fig. 4).

This study’s findings on improved seed quality from photobiomodulation with laser light were consistent with previously published reports. For instance, Podlesna et al. (2015) presented the positive effects of laser irradiation on the growth of white lupine and faba bean, while Hwida, Metwally & Lobna (2012) noted the improvements in seed quality of *Balanites aegyptiaca*. Furthermore, Taie et al. (2014) reported increases in the shoot length of *Sequoia sempervirens* from laser irradiation. Concerning the effects of He–Ne irradiation on the development of seeds, Metwally et al. (2013) reported a significant increase in the length of the *Celosia argentea* root, while Qiu et al. (2013) observed considerable growth of wheat seedlings. Podlesna et al. (2015) reported a significant difference in the root length of white lupine and faba bean from treatment with low doses of He–Ne laser light. These improvements are attributed to increased internal seed energy, which facilitated enzyme activity and encouraged cell division during seed germination (Tang et al., 2019).

However, further details are needed on the effects of different laser irradiation that cannot be explained (Samuilov & Garifullina, 2007). Coherent laser light beams excite electrons and promote biophoton and entropy emissions, thereby triggering an increase in the exposed material’s internal energy. The transient action of laser irradiation has also been implicated in stimulating various functional activities and higher plants’ resistance to biotic disease (Podleśny et al., 2012; Srećković et al., 2014; Tang et al., 2019).

Among other physical methods for increasing germination, considerable attention has been paid to low-intensity laser treatment, i.e., laser photobiomodulation (Ćwintal, Dziwulska-Hunek & Wilczek, 2010). This method of laser pre-sowing treatment of crop seeds can improve a plant’s physiological activity and increase crop yield (Kouchebagh et al., 2014b). Currently, this treatment is generally considered less harmful to the environment than other methods because it only modifies the seed’s physiological and natural biochemical processes (Hanafiah, Mohamad & Aziz, 2018; Harun, Hanafiah & Aziz, 2020). In addition, the availability of various laser wavelengths has grown in recent years. The relationship of laser beam wavelength to exposure time depends on different optical or thermal effects (Chen et al., 2005). Energy supply increases the potential power of seeds and influences the rate of biological activities during seed germination (Kouchebagh et al., 2014a).

Therefore, red light (He–Ne) laser irradiation can be used as a pre-germinative treatment of seeds to improve seed germination capacity and strengthen seedling vigor at the early development phase. At this phase, plants react positively to light at a wavelength range of 630 nm–650 nm (Hernandez-Aguilar, Dominguez-Pacheco & Cruz-Orea, 2015). González, Fortes & Herrera (2008) reported that laser power and irradiation time exert varying effects on seed germination. For example, the use of 1 mW of laser power increased the germination rate from 40% to 63%.

### Limitations and recommendations for future study

While previous studies have evaluated the effects of maize seedling performance in outdoor environments using various sources and intensity of lasers (Samuilov & Garifullina, 2007; Hernández-Aguilar et al., 2011; Srećković et al., 2014; Hasan et al., 2020a; Hasan et al., 2020b; Dziwulska-Hunek, Szymanek & Stadnik, 2020), few have reported physiological responses by using laser under these conditions (Asghar et al., 2016; Rózanowski et al., 2020). With increased understanding of these responses, production in controlled environments can be more effectively optimized. Our results indicate that seeds irradiated by green laser are significantly improved by acclimation responses at various scales. The present study provides a foundation for future research to elucidate how Nd:YAG laser radiation intensity and quality can be used alongside period of the irradiation to optimize seedling growth and quality for the efficient use of energy.

The obtained results may be utilized in the context of agrotechnical treatment applicable in ecofriendly agriculture, with the view of enhancing the sowing material. On the other hand, the limitation of this study is to irradiate hundreds seeds in same time, but depending on the development in the science of laser technology, it could be said that this problem has been addressed by using laser arrays which means hundreds of laser beams could irradiate the seeds at the same time. One of unique features of laser is the ease of automation for any process so we can irradiate a target by hundreds or thousands of laser beams within the same time or period.

Recently the use of physical methods for plant growth stimulation is getting more popular due to the less harmful influence on the environment. Physical factors can be used to get positive biological changes in crop plants without affecting the ecology. One of these physical methods is the laser light which this study focused to evaluate the effects of two different wavelengths by different intensity for maize field performance in outdoor environments.

On the other side, LED is the other physical method which is the main source for Vertical Farms to grow organic, pesticide free, climate-controlled food inside indoor environments. The benefits of Vertical Farms use less water, less energy and enable people to grow food underground or indoors year-round in any climate. However, there are no study conducted to combine both laser and LED technologies because it needs to high numbers of factors to be compared. Therefore, we recommended for future study to conduct the effect of Nd:YAG laser irradiation on seeds along with growing them indoor control-conditions under one of LED source.

## Conclusions

This study has demonstrated how pretreatment of seeds with laser irradiation at 632.8 nm and 532 nm enhances the emergence, growth, and development of *Zea mays* L. The laser pre-sowing seed treatment was observed to enhance the emergence of maize seeds. The emergence percentage, mean emergence time, and vigor index values significantly increased in plants irradiated by the green laser for 105 s. The seedling length, stem diameter, shoot fresh weight, shoot dry weight, days of anthesis, and silking values, as well as seed quality parameters, were found to be statistically higher in plants treated by a green laser. The Nd:YAG laser pre-sowing seed treatment showed promising efficiency compared to the He–Ne laser and can enhance maize productivity. Photobiomodulation of maize can be useful not only for consumption but also for protecting the environment and production of alternative energy (biofuels, biogas). Therefore, further studies on the impact of laser photobiomodulation on plant processes may contribute to increasing production and improving food quality and the effective protection and shaping of the natural environment, especially in regions impacted by industrial uses. Furthermore, a study of the mechanism underlying phytohormones should be conducted using the optimum He–Ne and Nd:YAG laser pretreatment.

##  Supplemental Information

10.7717/peerj.10614/supp-1 Supplemental Information 1 Experimental Raw Data

10.7717/peerj.2399/supp-2 Supplemental Information 1 GenStat Software Report for Analysed Data

## Additional Information and Declarations

### Data Availability

The following information was supplied regarding data availability: Raw data are available as Supplemental Files.
