# Supplementary material for: Comparison of the effects of two laser photobiomodulation techniques on bio-physical properties of Zea mays L. seeds
Source: PeerJ. 2021 Jan 15;9:e10614. doi: 10.7717/peerj.10614 (PMC7812920; doi:10.7717/peerj.10614)
Supplement: Supplemental Information 1 [file peerj-09-10614-s002.rtf]

GenStat Release 12.1 ( PC/Windows Vista) 23 March 2020 02:05:15
Copyright 2009, VSN International Ltd.  
Registered to: The NULL Corporation
 
 	________________________________________
 
 	GenStat Twelfth Edition
 	GenStat Procedure Library Release PL20.1
 	________________________________________
 
   1  %CD 'C:/Users/Mohammed/Documents'
   2  "Data taken from unsaved spreadsheet: New Data;1"
   3  %PostMessage 1129; 0; 82801936 "Sheet Update Completed"
   4  "Data taken from unsaved spreadsheet: New Data;1"
   5  %PostMessage 1129; 0; 82801936 "Sheet Update Completed"
   6  "Data taken from unsaved spreadsheet: New Data;1"
   7  DELETE [REDEFINE=yes] C1,C2
   8  UNITS [NVALUES=*]
   9  TEXT [NVALUES=51] C1
  10  READ C1
 
	 Identifier	 Minimum	 Mean	 Maximum	 Values	 Missing	 
	C1	 	 	 	 51	 0	 
 
  17  VARIATE [NVALUES=51] C2
  18  READ C2
 
	 Identifier	 Minimum	 Mean	 Maximum	 Values	 Missing	 
	C2	 1.000	 2.000	 3.000	 51	 0	 
 
  21  RESTRICT C1,C2
  22
  23  %PostMessage 1129; 0; 82801936 "Sheet Update Completed"
  24  "Data taken from unsaved spreadsheet: New Data;1"
  25  UNITS [NVALUES=*]
  26
  27  %PostMessage 1129; 0; 82801936 "Sheet Update Completed"
  28  "Data taken from unsaved spreadsheet: New Data;1"
  29  DELETE [REDEFINE=yes] Treatments,REP,C3,C4,C5,C6,C7,C8,C9,C10,C11,C12,C13,\
  30  C14,C15,C16,C17
  31  UNITS [NVALUES=*]
  32  TEXT [NVALUES=51] Treatments
  33  READ Treatments
 
	 Identifier	 Minimum	 Mean	 Maximum	 Values	 Missing	 
	Treatments	 	 	 	 51	 0	 
 
  40  VARIATE [NVALUES=51] REP
  41  READ REP
 
	 Identifier	 Minimum	 Mean	 Maximum	 Values	 Missing	 
	REP	 1.000	 2.000	 3.000	 51	 0	 
 
  44  VARIATE [NVALUES=51] C3
  45  READ C3
 
	 Identifier	 Minimum	 Mean	 Maximum	 Values	 Missing	 
	C3	 61.90	 78.23	 92.37	 51	 0	 
 
  54  VARIATE [NVALUES=51] C4
  55  READ C4
 
	 Identifier	 Minimum	 Mean	 Maximum	 Values	 Missing	 
	C4	 3.953	 5.882	 7.617	 51	 0	 
 
  68  VARIATE [NVALUES=51] C5
  69  READ C5
 
	 Identifier	 Minimum	 Mean	 Maximum	 Values	 Missing	 
	C5	 22.24	 41.86	 60.96	 51	 0	 
 
  82  VARIATE [NVALUES=51] C6
  83  READ C6
 
	 Identifier	 Minimum	 Mean	 Maximum	 Values	 Missing	 
	C6	 36.33	 48.29	 64.40	 51	 0	 
 
  94  VARIATE [NVALUES=51] C7
  95  READ C7
 
	 Identifier	 Minimum	 Mean	 Maximum	 Values	 Missing	 
	C7	 122.3	 263.5	 356.3	 51	 0	 
 
 101  VARIATE [NVALUES=51] C8
 102  READ C8
 
	 Identifier	 Minimum	 Mean	 Maximum	 Values	 Missing	 
	C8	 11.30	 19.79	 24.70	 51	 0	 
 
 107  VARIATE [NVALUES=51] C9
 108  READ C9
 
	 Identifier	 Minimum	 Mean	 Maximum	 Values	 Missing	 
	C9	 6.600	 7.635	 8.900	 51	 0	 
 
 112  VARIATE [NVALUES=51] C10
 113  READ C10
 
	 Identifier	 Minimum	 Mean	 Maximum	 Values	 Missing	 
	C10	 0.5320	 2.678	 5.102	 51	 0	 
 
 119  VARIATE [NVALUES=51] C11
 120  READ C11
 
	 Identifier	 Minimum	 Mean	 Maximum	 Values	 Missing	 
	C11	 0.02000	 0.5624	 1.314	 51	 0	 
 
 126  VARIATE [NVALUES=51] C12
 127  READ C12
 
	 Identifier	 Minimum	 Mean	 Maximum	 Values	 Missing	 
	C12	 1.200	 2.426	 3.900	 51	 0	 
 
 131  VARIATE [NVALUES=51] C13
 132  READ C13
 
	 Identifier	 Minimum	 Mean	 Maximum	 Values	 Missing	 
	C13	 56.20	 60.56	 63.50	 51	 0	 
 
 137  VARIATE [NVALUES=51] C14
 138  READ C14
 
	 Identifier	 Minimum	 Mean	 Maximum	 Values	 Missing	 
	C14	 60.30	 63.37	 68.60	 51	 0	 
 
 143  VARIATE [NVALUES=51] C15
 144  READ C15
 
	 Identifier	 Minimum	 Mean	 Maximum	 Values	 Missing	 
	C15	 13.16	 15.80	 18.27	 51	 0	 
 
 149  VARIATE [NVALUES=51] C16
 150  READ C16
 
	 Identifier	 Minimum	 Mean	 Maximum	 Values	 Missing	 
	C16	 3.080	 5.126	 6.840	 51	 0	 
 
 155  VARIATE [NVALUES=51] C17
 156  READ C17
 
	 Identifier	 Minimum	 Mean	 Maximum	 Values	 Missing	 
	C17	 62.52	 67.81	 73.90	 51	 0	 
 
 161
 162  %PostMessage 1129; 0; 82801936 "Sheet Update Completed"
 163  "Data taken from unsaved spreadsheet: New Data;1"
 164  DELETE [REDEFINE=yes] Emergence_%
 165  UNITS [NVALUES=*]
 166  VARIATE [NVALUES=51] Emergence_%
 167  READ Emergence_%
 
	 Identifier	 Minimum	 Mean	 Maximum	 Values	 Missing	 
	Emergence_%	 61.90	 78.23	 92.37	 51	 0	 
 
 176
 177  %PostMessage 1129; 0; 82801936 "Sheet Update Completed"
 178  "Data taken from unsaved spreadsheet: New Data;1"
 179  DELETE [REDEFINE=yes] Emergence_index
 180  UNITS [NVALUES=*]
 181  VARIATE [NVALUES=51] Emergence_index
 182  READ Emergence_index
 
	 Identifier	 Minimum	 Mean	 Maximum	 Values	 Missing	 
	Emergence_index	 3.953	 5.882	 7.617	 51	 0	 
 
 195
 196  %PostMessage 1129; 0; 82801936 "Sheet Update Completed"
 197  "Data taken from unsaved spreadsheet: New Data;1"
 198  DELETE [REDEFINE=yes] Speed_emergence
 199  UNITS [NVALUES=*]
 200  VARIATE [NVALUES=51] Speed_emergence
 201  READ Speed_emergence
 
	 Identifier	 Minimum	 Mean	 Maximum	 Values	 Missing	 
	Speed_emergence	 22.24	 41.86	 60.96	 51	 0	 
 
 214
 215  %PostMessage 1129; 0; 82801936 "Sheet Update Completed"
 216  "Data taken from unsaved spreadsheet: New Data;1"
 217  DELETE [REDEFINE=yes] Mean_emergence_time
 218  UNITS [NVALUES=*]
 219  VARIATE [NVALUES=51] Mean_emergence_time
 220  READ Mean_emergence_time
 
	 Identifier	 Minimum	 Mean	 Maximum	 Values	 Missing	 
	Mean_emergence_time	 36.33	 48.29	 64.40	 51	 0	 
 
 231
 232  %PostMessage 1129; 0; 82801936 "Sheet Update Completed"
 233  "Data taken from unsaved spreadsheet: New Data;1"
 234  DELETE [REDEFINE=yes] Vigour_index
 235  UNITS [NVALUES=*]
 236  VARIATE [NVALUES=51] Vigour_index
 237  READ Vigour_index
 
	 Identifier	 Minimum	 Mean	 Maximum	 Values	 Missing	 
	Vigour_index	 122.3	 263.5	 356.3	 51	 0	 
 
 243
 244  %PostMessage 1129; 0; 82801936 "Sheet Update Completed"
 245  "Data taken from unsaved spreadsheet: New Data;1"
 246  DELETE [REDEFINE=yes] Seedling_length
 247  UNITS [NVALUES=*]
 248  VARIATE [NVALUES=51] Seedling_length
 249  READ Seedling_length
 
	 Identifier	 Minimum	 Mean	 Maximum	 Values	 Missing	 
	Seedling_length	 11.30	 19.79	 24.70	 51	 0	 
 
 254
 255  %PostMessage 1129; 0; 82801936 "Sheet Update Completed"
 256  "Data taken from unsaved spreadsheet: New Data;1"
 257  DELETE [REDEFINE=yes] Leaves_number
 258  UNITS [NVALUES=*]
 259  VARIATE [NVALUES=51] Leaves_number
 260  READ Leaves_number
 
	 Identifier	 Minimum	 Mean	 Maximum	 Values	 Missing	 
	Leaves_number	 6.600	 7.635	 8.900	 51	 0	 
 
 264
 265  %PostMessage 1129; 0; 82801936 "Sheet Update Completed"
 266  "Data taken from unsaved spreadsheet: New Data;1"
 267  DELETE [REDEFINE=yes] Fresh_weight
 268  UNITS [NVALUES=*]
 269  VARIATE [NVALUES=51] Fresh_weight
 270  READ Fresh_weight
 
	 Identifier	 Minimum	 Mean	 Maximum	 Values	 Missing	 
	Fresh_weight	 0.5320	 2.678	 5.102	 51	 0	 
 
 276
 277  %PostMessage 1129; 0; 82801936 "Sheet Update Completed"
 278  "Data taken from unsaved spreadsheet: New Data;1"
 279  DELETE [REDEFINE=yes] Dry_weight
 280  UNITS [NVALUES=*]
 281  VARIATE [NVALUES=51] Dry_weight
 282  READ Dry_weight
 
	 Identifier	 Minimum	 Mean	 Maximum	 Values	 Missing	 
	Dry_weight	 0.02000	 0.5624	 1.314	 51	 0	 
 
 288
 289  %PostMessage 1129; 0; 82801936 "Sheet Update Completed"
 290  "Data taken from unsaved spreadsheet: New Data;1"
 291  DELETE [REDEFINE=yes] Stem_diameter
 292  UNITS [NVALUES=*]
 293  VARIATE [NVALUES=51] Stem_diameter
 294  READ Stem_diameter
 
	 Identifier	 Minimum	 Mean	 Maximum	 Values	 Missing	 
	Stem_diameter	 1.200	 2.426	 3.900	 51	 0	 
 
 298
 299  %PostMessage 1129; 0; 82801936 "Sheet Update Completed"
 300  "Data taken from unsaved spreadsheet: New Data;1"
 301  DELETE [REDEFINE=yes] Days_to_Anthesis
 302  UNITS [NVALUES=*]
 303  VARIATE [NVALUES=51] Days_to_Anthesis
 304  READ Days_to_Anthesis
 
	 Identifier	 Minimum	 Mean	 Maximum	 Values	 Missing	 
	Days_to_Anthesis	 56.20	 60.56	 63.50	 51	 0	 
 
 309
 310  %PostMessage 1129; 0; 82801936 "Sheet Update Completed"
 311  "Data taken from unsaved spreadsheet: New Data;1"
 312  DELETE [REDEFINE=yes] Days_to_Silking
 313  UNITS [NVALUES=*]
 314  VARIATE [NVALUES=51] Days_to_Silking
 315  READ Days_to_Silking
 
	 Identifier	 Minimum	 Mean	 Maximum	 Values	 Missing	 
	Days_to_Silking	 60.30	 63.37	 68.60	 51	 0	 
 
 320
 321  %PostMessage 1129; 0; 82801936 "Sheet Update Completed"
 322  "Data taken from unsaved spreadsheet: New Data;1"
 323  DELETE [REDEFINE=yes] Protein_content
 324  UNITS [NVALUES=*]
 325  VARIATE [NVALUES=51] Protein_content
 326  READ Protein_content
 
	 Identifier	 Minimum	 Mean	 Maximum	 Values	 Missing	 
	Protein_content	 13.16	 15.80	 18.27	 51	 0	 
 
 331
 332  %PostMessage 1129; 0; 82801936 "Sheet Update Completed"
 333  "Data taken from unsaved spreadsheet: New Data;1"
 334  DELETE [REDEFINE=yes] Oil_content
 335  UNITS [NVALUES=*]
 336  VARIATE [NVALUES=51] Oil_content
 337  READ Oil_content
 
	 Identifier	 Minimum	 Mean	 Maximum	 Values	 Missing	 
	Oil_content	 3.080	 5.126	 6.840	 51	 0	 
 
 342
 343  %PostMessage 1129; 0; 82801936 "Sheet Update Completed"
 344  "Data taken from unsaved spreadsheet: New Data;1"
 345  DELETE [REDEFINE=yes] Treatments,REP,Starch_content
 346  UNITS [NVALUES=*]
 347  FACTOR [MODIFY=yes; NVALUES=51; LEVELS=17; LABELS=!t('Control','GT1S1',\
 348  'GT1S2','GT2S1','GT2S2','GT3S1','GT3S2','GT4S1','GT4S2','RT1S1','RT1S2',\
 349  'RT2S1','RT2S2','RT3S1','RT3S2','RT4S1','RT4S2'); REFERENCE=1] Treatments
 350  READ Treatments; FREPRESENTATION=ordinal
 
	 Identifier	 Values	 Missing	 Levels
	Treatments	 51	 0	 17
 
 353  FACTOR [MODIFY=yes; NVALUES=51; LEVELS=3; REFERENCE=1] REP
 354  READ REP; FREPRESENTATION=ordinal
 
	 Identifier	 Values	 Missing	 Levels
	REP	 51	 0	 3
 
 357  VARIATE [NVALUES=51] Starch_content
 358  READ Starch_content
 
	 Identifier	 Minimum	 Mean	 Maximum	 Values	 Missing	 
	Starch_content	 62.52	 67.81	 73.90	 51	 0	 
 
 363
 364  %PostMessage 1129; 0; 82801936 "Sheet Update Completed"
 365  "General Analysis of Variance."
 366  BLOCK REP
 367  TREATMENTS Treatments
 368  COVARIATE "No Covariate"
 369  ANOVA [PRINT=aovtable,information,means,%cv; FACT=32; CONTRASTS=7; PCONTRASTS=7; FPROB=yes;\
 370   PSE=diff,lsd,means; LSDLEVEL=1] Emergence_%

Analysis of variance
 
Variate: Emergence_%
 
Source of variation	d.f.	s.s.	m.s.	v.r.	F pr.
 
REP stratum	2	 2.104	 1.052	 1.03	 
 
REP.*Units* stratum
Treatments	16	 4251.324	 265.708	 261.11	<.001
Residual	32	 32.563	 1.018	 	 
 
Total	50	 4285.991	 	 	 
 
 
Message: the following units have large residuals.
 
REP 2 *units* 5	   1.91	 s.e.   0.80
REP 2 *units* 12	   1.91	 s.e.   0.80
 
 
Tables of means
 
Variate: Emergence_%
 
Grand mean  78.23 
 
	Treatments	 Control	 GT1S1	 GT1S2	 GT2S1	 GT2S2	 GT3S1	 GT3S2
		 62.50	 79.17	 87.50	 87.83	 83.33	 75.00	 79.17
	 
	Treatments	 GT4S1	 GT4S2	 RT1S1	 RT1S2	 RT2S1	 RT2S2	 RT3S1
		 75.00	 91.67	 62.50	 83.67	 70.83	 87.50	 79.17
	 
	Treatments	 RT3S2	 RT4S1	 RT4S2	 	 	 	 
		 87.50	 75.00	 62.50	 	 	 	 
 
 
Standard errors of means
 
Table	Treatments	 
rep.	 3	 
d.f.	 32	 
e.s.e.	 0.582	 
 
 
 
Standard errors of differences of means
 
Table	Treatments	 
rep.	 3	 
d.f.	 32	 
s.e.d.	 0.824	 
 
 
 
Least significant differences of means (1% level)
 
Table	Treatments	 
rep.	 3	 
d.f.	 32	 
l.s.d.	 2.256	 
 
 
 
Stratum standard errors and coefficients of variation
 
Variate: Emergence_%
 
Stratum	d.f.	s.e.	cv%
REP	 2	 0.249	 0.3
REP.*Units*	 32	 1.009	 1.3
 
 371  "General Analysis of Variance."
 372  BLOCK REP
 373  TREATMENTS Treatments
 374  COVARIATE "No Covariate"
 375  ANOVA [PRINT=aovtable,information,means,%cv; FACT=32; CONTRASTS=7; PCONTRASTS=7; FPROB=yes;\
 376   PSE=diff,lsd,means; LSDLEVEL=1] Emergence_index

Analysis of variance
 
Variate: Emergence_index
 
Source of variation	d.f.	s.s.	m.s.	v.r.	F pr.
 
REP stratum	2	 0.6444	 0.3222	 1.29	 
 
REP.*Units* stratum
Treatments	16	 24.2941	 1.5184	 6.10	<.001
Residual	32	 7.9626	 0.2488	 	 
 
Total	50	 32.9011	 	 	 
 
 
Message: the following units have large residuals.
 
REP 3 *units* 6	   0.851	 s.e.   0.395
REP 3 *units* 15	   0.851	 s.e.   0.395
 
 
Tables of means
 
Variate: Emergence_index
 
Grand mean  5.882 
 
	Treatments	 Control	 GT1S1	 GT1S2	 GT2S1	 GT2S2	 GT3S1	 GT3S2
		 4.583	 5.667	 6.333	 6.000	 6.417	 5.333	 6.000
	 
	Treatments	 GT4S1	 GT4S2	 RT1S1	 RT1S2	 RT2S1	 RT2S2	 RT3S1
		 5.083	 4.583	 6.333	 7.167	 6.083	 5.417	 6.667
	 
	Treatments	 RT3S2	 RT4S1	 RT4S2	 	 	 	 
		 6.583	 5.833	 5.917	 	 	 	 
 
 
Standard errors of means
 
Table	Treatments	 
rep.	 3	 
d.f.	 32	 
e.s.e.	 0.2880	 
 
 
 
Standard errors of differences of means
 
Table	Treatments	 
rep.	 3	 
d.f.	 32	 
s.e.d.	 0.4073	 
 
 
 
Least significant differences of means (1% level)
 
Table	Treatments	 
rep.	 3	 
d.f.	 32	 
l.s.d.	 1.1154	 
 
 
 
Stratum standard errors and coefficients of variation
 
Variate: Emergence_index
 
Stratum	d.f.	s.e.	cv%
REP	 2	 0.1377	 2.3
REP.*Units*	 32	 0.4988	 8.5
 
 377  "General Analysis of Variance."
 378  BLOCK REP
 379  TREATMENTS Treatments
 380  COVARIATE "No Covariate"
 381  ANOVA [PRINT=aovtable,information,means,%cv; FACT=32; CONTRASTS=7; PCONTRASTS=7; FPROB=yes;\
 382   PSE=diff,lsd,means; LSDLEVEL=1] Speed_emergence

Analysis of variance
 
Variate: Speed_emergence
 
Source of variation	d.f.	s.s.	m.s.	v.r.	F pr.
 
REP stratum	2	 3.1109	 1.5555	 1.59	 
 
REP.*Units* stratum
Treatments	16	 4548.9134	 284.3071	 290.90	<.001
Residual	32	 31.2751	 0.9773	 	 
 
Total	50	 4583.2995	 	 	 
 
 
Message: the following units have large residuals.
 
REP 1 *units* 17	   -2.44	 s.e.   0.78
 
 
Tables of means
 
Variate: Speed_emergence
 
Grand mean  41.86 
 
	Treatments	 Control	 GT1S1	 GT1S2	 GT2S1	 GT2S2	 GT3S1	 GT3S2
		 38.60	 34.68	 40.52	 29.29	 51.60	 31.78	 45.84
	 
	Treatments	 GT4S1	 GT4S2	 RT1S1	 RT1S2	 RT2S1	 RT2S2	 RT3S1
		 23.44	 31.33	 48.80	 60.36	 40.40	 42.71	 51.76
	 
	Treatments	 RT3S2	 RT4S1	 RT4S2	 	 	 	 
		 40.26	 48.95	 51.22	 	 	 	 
 
 
Standard errors of means
 
Table	Treatments	 
rep.	 3	 
d.f.	 32	 
e.s.e.	 0.571	 
 
 
 
Standard errors of differences of means
 
Table	Treatments	 
rep.	 3	 
d.f.	 32	 
s.e.d.	 0.807	 
 
 
 
Least significant differences of means (1% level)
 
Table	Treatments	 
rep.	 3	 
d.f.	 32	 
l.s.d.	 2.210	 
 
 
 
Stratum standard errors and coefficients of variation
 
Variate: Speed_emergence
 
Stratum	d.f.	s.e.	cv%
REP	 2	 0.302	 0.7
REP.*Units*	 32	 0.989	 2.4
 
 383  "General Analysis of Variance."
 384  BLOCK REP
 385  TREATMENTS Treatments
 386  COVARIATE "No Covariate"
 387  ANOVA [PRINT=aovtable,information,means,%cv; FACT=32; CONTRASTS=7; PCONTRASTS=7; FPROB=yes;\
 388   PSE=diff,lsd,means; LSDLEVEL=1] Mean_emergence_time

Analysis of variance
 
Variate: Mean_emergence_time
 
Source of variation	d.f.	s.s.	m.s.	v.r.	F pr.
 
REP stratum	2	 6.155	 3.078	 1.86	 
 
REP.*Units* stratum
Treatments	16	 2236.962	 139.810	 84.43	<.001
Residual	32	 52.993	 1.656	 	 
 
Total	50	 2296.110	 	 	 
 
 
Message: the following units have large residuals.
 
REP 3 *units* 6	   -2.35	 s.e.   1.02
REP 3 *units* 14	   -2.35	 s.e.   1.02
 
 
Tables of means
 
Variate: Mean_emergence_time
 
Grand mean  48.29 
 
	Treatments	 Control	 GT1S1	 GT1S2	 GT2S1	 GT2S2	 GT3S1	 GT3S2
		 45.33	 43.91	 49.58	 44.33	 54.71	 39.00	 50.07
	 
	Treatments	 GT4S1	 GT4S2	 RT1S1	 RT1S2	 RT2S1	 RT2S2	 RT3S1
		 38.29	 37.00	 53.00	 62.52	 48.15	 45.86	 55.94
	 
	Treatments	 RT3S2	 RT4S1	 RT4S2	 	 	 	 
		 46.85	 54.25	 52.24	 	 	 	 
 
 
Standard errors of means
 
Table	Treatments	 
rep.	 3	 
d.f.	 32	 
e.s.e.	 0.743	 
 
 
 
Standard errors of differences of means
 
Table	Treatments	 
rep.	 3	 
d.f.	 32	 
s.e.d.	 1.051	 
 
 
 
Least significant differences of means (1% level)
 
Table	Treatments	 
rep.	 3	 
d.f.	 32	 
l.s.d.	 2.877	 
 
 
 
Stratum standard errors and coefficients of variation
 
Variate: Mean_emergence_time
 
Stratum	d.f.	s.e.	cv%
REP	 2	 0.425	 0.9
REP.*Units*	 32	 1.287	 2.7
 
 389  "General Analysis of Variance."
 390  BLOCK REP
 391  TREATMENTS Treatments
 392  COVARIATE "No Covariate"
 393  ANOVA [PRINT=aovtable,information,means,%cv; FACT=32; CONTRASTS=7; PCONTRASTS=7; FPROB=yes;\
 394   PSE=diff,lsd,means; LSDLEVEL=1] Vigour_index

Analysis of variance
 
Variate: Vigour_index
 
Source of variation	d.f.	s.s.	m.s.	v.r.	F pr.
 
REP stratum	2	 0.520	 0.260	 0.24	 
 
REP.*Units* stratum
Treatments	16	 220916.824	 13807.301	 12676.98	<.001
Residual	32	 34.853	 1.089	 	 
 
Total	50	 220952.196	 	 	 
 
 
Tables of means
 
Variate: Vigour_index
 
Grand mean  263.54 
 
	Treatments	 Control	 GT1S1	 GT1S2	 GT2S1	 GT2S2	 GT3S1	 GT3S2
		 188.00	 224.40	 280.80	 217.80	 343.40	 194.00	 214.00
	 
	Treatments	 GT4S1	 GT4S2	 RT1S1	 RT1S2	 RT2S1	 RT2S2	 RT3S1
		 123.20	 351.00	 355.20	 272.00	 226.20	 294.00	 336.60
	 
	Treatments	 RT3S2	 RT4S1	 RT4S2	 	 	 	 
		 252.20	 345.60	 261.80	 	 	 	 
 
 
Standard errors of means
 
Table	Treatments	 
rep.	 3	 
d.f.	 32	 
e.s.e.	 0.603	 
 
 
 
Standard errors of differences of means
 
Table	Treatments	 
rep.	 3	 
d.f.	 32	 
s.e.d.	 0.852	 
 
 
 
Least significant differences of means (1% level)
 
Table	Treatments	 
rep.	 3	 
d.f.	 32	 
l.s.d.	 2.334	 
 
 
 
Stratum standard errors and coefficients of variation
 
Variate: Vigour_index
 
Stratum	d.f.	s.e.	cv%
REP	 2	 0.124	 0.0
REP.*Units*	 32	 1.044	 0.4
 
 395  "General Analysis of Variance."
 396  BLOCK REP
 397  TREATMENTS Treatments
 398  COVARIATE "No Covariate"
 399  ANOVA [PRINT=aovtable,information,means,%cv; FACT=32; CONTRASTS=7; PCONTRASTS=7; FPROB=yes;\
 400   PSE=diff,lsd,means; LSDLEVEL=1] Seedling_length

Analysis of variance
 
Variate: Seedling_length
 
Source of variation	d.f.	s.s.	m.s.	v.r.	F pr.
 
REP stratum	2	 5.297	 2.649	 1.24	 
 
REP.*Units* stratum
Treatments	16	 352.172	 22.011	 10.32	<.001
Residual	32	 68.257	 2.133	 	 
 
Total	50	 425.726	 	 	 
 
 
Message: the following units have large residuals.
 
REP 2 *units* 1	   -2.76	 s.e.   1.16
REP 2 *units* 9	   -2.76	 s.e.   1.16
 
 
Tables of means
 
Variate: Seedling_length
 
Grand mean  19.79 
 
	Treatments	 Control	 GT1S1	 GT1S2	 GT2S1	 GT2S2	 GT3S1	 GT3S2
		 18.80	 20.40	 21.60	 24.20	 20.20	 19.73	 23.40
	 
	Treatments	 GT4S1	 GT4S2	 RT1S1	 RT1S2	 RT2S1	 RT2S2	 RT3S1
		 17.60	 21.40	 22.20	 13.60	 17.40	 20.08	 19.47
	 
	Treatments	 RT3S2	 RT4S1	 RT4S2	 	 	 	 
		 19.40	 21.60	 15.40	 	 	 	 
 
 
Standard errors of means
 
Table	Treatments	 
rep.	 3	 
d.f.	 32	 
e.s.e.	 0.843	 
 
 
 
Standard errors of differences of means
 
Table	Treatments	 
rep.	 3	 
d.f.	 32	 
s.e.d.	 1.192	 
 
 
 
Least significant differences of means (1% level)
 
Table	Treatments	 
rep.	 3	 
d.f.	 32	 
l.s.d.	 3.266	 
 
 
 
Stratum standard errors and coefficients of variation
 
Variate: Seedling_length
 
Stratum	d.f.	s.e.	cv%
REP	 2	 0.395	 2.0
REP.*Units*	 32	 1.460	 7.4
 
 401  "General Analysis of Variance."
 402  BLOCK REP
 403  TREATMENTS Treatments
 404  COVARIATE "No Covariate"
 405  ANOVA [PRINT=aovtable,information,means,%cv; FACT=32; CONTRASTS=7; PCONTRASTS=7; FPROB=yes;\
 406   PSE=diff,lsd,means; LSDLEVEL=1] Leaves_number

Analysis of variance
 
Variate: Leaves_number
 
Source of variation	d.f.	s.s.	m.s.	v.r.	F pr.
 
REP stratum	2	 0.1635	 0.0818	 0.56	 
 
REP.*Units* stratum
Treatments	16	 13.0165	 0.8135	 5.59	<.001
Residual	32	 4.6565	 0.1455	 	 
 
Total	50	 17.8365	 	 	 
 
 
Message: the following units have large residuals.
 
REP 1 *units* 3	   -0.759	 s.e.   0.302
REP 1 *units* 11	   -0.759	 s.e.   0.302
 
 
Tables of means
 
Variate: Leaves_number
 
Grand mean  7.635 
 
	Treatments	 Control	 GT1S1	 GT1S2	 GT2S1	 GT2S2	 GT3S1	 GT3S2
		 6.800	 7.200	 7.800	 8.200	 7.600	 8.000	 7.400
	 
	Treatments	 GT4S1	 GT4S2	 RT1S1	 RT1S2	 RT2S1	 RT2S2	 RT3S1
		 7.000	 7.800	 7.800	 6.800	 7.400	 8.400	 8.200
	 
	Treatments	 RT3S2	 RT4S1	 RT4S2	 	 	 	 
		 7.800	 8.400	 7.200	 	 	 	 
 
 
Standard errors of means
 
Table	Treatments	 
rep.	 3	 
d.f.	 32	 
e.s.e.	 0.2202	 
 
 
 
Standard errors of differences of means
 
Table	Treatments	 
rep.	 3	 
d.f.	 32	 
s.e.d.	 0.3115	 
 
 
 
Least significant differences of means (1% level)
 
Table	Treatments	 
rep.	 3	 
d.f.	 32	 
l.s.d.	 0.8529	 
 
 
 
Stratum standard errors and coefficients of variation
 
Variate: Leaves_number
 
Stratum	d.f.	s.e.	cv%
REP	 2	 0.0694	 0.9
REP.*Units*	 32	 0.3815	 5.0
 
 407  "General Analysis of Variance."
 408  BLOCK REP
 409  TREATMENTS Treatments
 410  COVARIATE "No Covariate"
 411  ANOVA [PRINT=aovtable,information,means,%cv; FACT=32; CONTRASTS=7; PCONTRASTS=7; FPROB=yes;\
 412   PSE=diff,lsd,means; LSDLEVEL=1] Fresh_weight

Analysis of variance
 
Variate: Fresh_weight
 
Source of variation	d.f.	s.s.	m.s.	v.r.	F pr.
 
REP stratum	2	 15.152	 7.576	 5.35	 
 
REP.*Units* stratum
Treatments	16	 14.646	 0.915	 0.65	 0.821
Residual	32	 45.282	 1.415	 	 
 
Total	50	 75.080	 	 	 
 
 
Message: the following units have large residuals.
 
REP 1 *units* 5	   2.25	 s.e.   0.94
REP 1 *units* 13	   2.25	 s.e.   0.94
 
 
Tables of means
 
Variate: Fresh_weight
 
Grand mean  2.68 
 
	Treatments	 Control	 GT1S1	 GT1S2	 GT2S1	 GT2S2	 GT3S1	 GT3S2
		 3.24	 2.65	 2.27	 4.10	 2.82	 2.89	 2.45
	 
	Treatments	 GT4S1	 GT4S2	 RT1S1	 RT1S2	 RT2S1	 RT2S2	 RT3S1
		 2.77	 2.66	 2.06	 2.73	 2.89	 2.55	 2.54
	 
	Treatments	 RT3S2	 RT4S1	 RT4S2	 	 	 	 
		 2.57	 2.95	 1.37	 	 	 	 
 
 
Standard errors of means
 
Table	Treatments	 
rep.	 3	 
d.f.	 32	 
e.s.e.	 0.687	 
 
 
 
Standard errors of differences of means
 
Table	Treatments	 
rep.	 3	 
d.f.	 32	 
s.e.d.	 0.971	 
 
 
 
Least significant differences of means (1% level)
 
Table	Treatments	 
rep.	 3	 
d.f.	 32	 
l.s.d.	 2.660	 
 
 
 
Stratum standard errors and coefficients of variation
 
Variate: Fresh_weight
 
Stratum	d.f.	s.e.	cv%
REP	 2	 0.668	 24.9
REP.*Units*	 32	 1.190	 44.4
 
 413  "General Analysis of Variance."
 414  BLOCK REP
 415  TREATMENTS Treatments
 416  COVARIATE "No Covariate"
 417  ANOVA [PRINT=aovtable,information,means,%cv; FACT=32; CONTRASTS=7; PCONTRASTS=7; FPROB=yes;\
 418   PSE=diff,lsd,means; LSDLEVEL=1] Dry_weight

Analysis of variance
 
Variate: Dry_weight
 
Source of variation	d.f.	s.s.	m.s.	v.r.	F pr.
 
REP stratum	2	 0.0301	 0.0151	 0.14	 
 
REP.*Units* stratum
Treatments	16	 0.6829	 0.0427	 0.40	 0.974
Residual	32	 3.4464	 0.1077	 	 
 
Total	50	 4.1594	 	 	 
 
 
Message: the following units have large residuals.
 
REP 1 *units* 3	   0.599	 s.e.   0.260
REP 1 *units* 11	   0.599	 s.e.   0.260
 
 
Tables of means
 
Variate: Dry_weight
 
Grand mean  0.562 
 
	Treatments	 Control	 GT1S1	 GT1S2	 GT2S1	 GT2S2	 GT3S1	 GT3S2
		 0.460	 0.780	 0.539	 0.714	 0.544	 0.548	 0.476
	 
	Treatments	 GT4S1	 GT4S2	 RT1S1	 RT1S2	 RT2S1	 RT2S2	 RT3S1
		 0.478	 0.522	 0.815	 0.717	 0.520	 0.528	 0.524
	 
	Treatments	 RT3S2	 RT4S1	 RT4S2	 	 	 	 
		 0.430	 0.544	 0.422	 	 	 	 
 
 
Standard errors of means
 
Table	Treatments	 
rep.	 3	 
d.f.	 32	 
e.s.e.	 0.1895	 
 
 
 
Standard errors of differences of means
 
Table	Treatments	 
rep.	 3	 
d.f.	 32	 
s.e.d.	 0.2680	 
 
 
 
Least significant differences of means (1% level)
 
Table	Treatments	 
rep.	 3	 
d.f.	 32	 
l.s.d.	 0.7338	 
 
 
 
Stratum standard errors and coefficients of variation
 
Variate: Dry_weight
 
Stratum	d.f.	s.e.	cv%
REP	 2	 0.0298	 5.3
REP.*Units*	 32	 0.3282	 58.4
 
 419  "General Analysis of Variance."
 420  BLOCK REP
 421  TREATMENTS Treatments
 422  COVARIATE "No Covariate"
 423  ANOVA [PRINT=aovtable,information,means,%cv; FACT=32; CONTRASTS=7; PCONTRASTS=7; FPROB=yes;\
 424   PSE=diff,lsd,means; LSDLEVEL=1] Stem_diameter

Analysis of variance
 
Variate: Stem_diameter
 
Source of variation	d.f.	s.s.	m.s.	v.r.	F pr.
 
REP stratum	2	 0.0268	 0.0134	 0.09	 
 
REP.*Units* stratum
Treatments	16	 9.3018	 0.5814	 3.71	<.001
Residual	32	 5.0182	 0.1568	 	 
 
Total	50	 14.3468	 	 	 
 
 
Message: the following units have large residuals.
 
REP 3 *units* 9	   0.871	 s.e.   0.314
 
 
Tables of means
 
Variate: Stem_diameter
 
Grand mean  2.426 
 
	Treatments	 Control	 GT1S1	 GT1S2	 GT2S1	 GT2S2	 GT3S1	 GT3S2
		 2.900	 2.100	 3.400	 2.500	 2.200	 1.500	 2.500
	 
	Treatments	 GT4S1	 GT4S2	 RT1S1	 RT1S2	 RT2S1	 RT2S2	 RT3S1
		 2.600	 2.900	 2.700	 2.200	 2.800	 2.400	 2.000
	 
	Treatments	 RT3S2	 RT4S1	 RT4S2	 	 	 	 
		 2.100	 2.300	 2.150	 	 	 	 
 
 
Standard errors of means
 
Table	Treatments	 
rep.	 3	 
d.f.	 32	 
e.s.e.	 0.2286	 
 
 
 
Standard errors of differences of means
 
Table	Treatments	 
rep.	 3	 
d.f.	 32	 
s.e.d.	 0.3233	 
 
 
 
Least significant differences of means (1% level)
 
Table	Treatments	 
rep.	 3	 
d.f.	 32	 
l.s.d.	 0.8855	 
 
 
 
Stratum standard errors and coefficients of variation
 
Variate: Stem_diameter
 
Stratum	d.f.	s.e.	cv%
REP	 2	 0.0281	 1.2
REP.*Units*	 32	 0.3960	 16.3
 
 425  "General Analysis of Variance."
 426  BLOCK REP
 427  TREATMENTS Treatments
 428  COVARIATE "No Covariate"
 429  ANOVA [PRINT=aovtable,information,means,%cv; FACT=32; CONTRASTS=7; PCONTRASTS=7; FPROB=yes;\
 430   PSE=diff,lsd,means; LSDLEVEL=1] Days_to_Anthesis

Analysis of variance
 
Variate: Days_to_Anthesis
 
Source of variation	d.f.	s.s.	m.s.	v.r.	F pr.
 
REP stratum	2	 14.906	 7.453	 6.69	 
 
REP.*Units* stratum
Treatments	16	 102.004	 6.375	 5.72	<.001
Residual	32	 35.667	 1.115	 	 
 
Total	50	 152.577	 	 	 
 
 
Message: the following units have large residuals.
 
REP 1 *units* 13	   -2.52	 s.e.   0.84
REP 3 *units* 3	   1.86	 s.e.   0.84
REP 3 *units* 13	   1.86	 s.e.   0.84
 
 
Tables of means
 
Variate: Days_to_Anthesis
 
Grand mean  60.56 
 
	Treatments	 Control	 GT1S1	 GT1S2	 GT2S1	 GT2S2	 GT3S1	 GT3S2
		 61.40	 60.40	 59.32	 58.30	 62.30	 60.74	 61.40
	 
	Treatments	 GT4S1	 GT4S2	 RT1S1	 RT1S2	 RT2S1	 RT2S2	 RT3S1
		 57.40	 61.94	 58.90	 61.30	 60.70	 60.10	 59.65
	 
	Treatments	 RT3S2	 RT4S1	 RT4S2	 	 	 	 
		 61.40	 61.50	 62.80	 	 	 	 
 
 
Standard errors of means
 
Table	Treatments	 
rep.	 3	 
d.f.	 32	 
e.s.e.	 0.610	 
 
 
 
Standard errors of differences of means
 
Table	Treatments	 
rep.	 3	 
d.f.	 32	 
s.e.d.	 0.862	 
 
 
 
Least significant differences of means (1% level)
 
Table	Treatments	 
rep.	 3	 
d.f.	 32	 
l.s.d.	 2.361	 
 
 
 
Stratum standard errors and coefficients of variation
 
Variate: Days_to_Anthesis
 
Stratum	d.f.	s.e.	cv%
REP	 2	 0.662	 1.1
REP.*Units*	 32	 1.056	 1.7
 
 431  "General Analysis of Variance."
 432  BLOCK REP
 433  TREATMENTS Treatments
 434  COVARIATE "No Covariate"
 435  ANOVA [PRINT=aovtable,information,means,%cv; FACT=32; CONTRASTS=7; PCONTRASTS=7; FPROB=yes;\
 436   PSE=diff,lsd,means; LSDLEVEL=1] Days_to_Silking

Analysis of variance
 
Variate: Days_to_Silking
 
Source of variation	d.f.	s.s.	m.s.	v.r.	F pr.
 
REP stratum	2	 5.227	 2.613	 1.40	 
 
REP.*Units* stratum
Treatments	16	 63.631	 3.977	 2.13	 0.034
Residual	32	 59.745	 1.867	 	 
 
Total	50	 128.603	 	 	 
 
 
Message: the following units have large residuals.
 
REP 3 *units* 8	   2.76	 s.e.   1.08
 
 
Tables of means
 
Variate: Days_to_Silking
 
Grand mean  63.37 
 
	Treatments	 Control	 GT1S1	 GT1S2	 GT2S1	 GT2S2	 GT3S1	 GT3S2
		 63.00	 61.50	 63.90	 63.30	 62.70	 62.25	 64.00
	 
	Treatments	 GT4S1	 GT4S2	 RT1S1	 RT1S2	 RT2S1	 RT2S2	 RT3S1
		 64.10	 65.40	 61.50	 63.90	 63.30	 62.70	 62.25
	 
	Treatments	 RT3S2	 RT4S1	 RT4S2	 	 	 	 
		 64.00	 64.10	 65.40	 	 	 	 
 
 
Standard errors of means
 
Table	Treatments	 
rep.	 3	 
d.f.	 32	 
e.s.e.	 0.789	 
 
 
 
Standard errors of differences of means
 
Table	Treatments	 
rep.	 3	 
d.f.	 32	 
s.e.d.	 1.116	 
 
 
 
Least significant differences of means (1% level)
 
Table	Treatments	 
rep.	 3	 
d.f.	 32	 
l.s.d.	 3.055	 
 
 
 
Stratum standard errors and coefficients of variation
 
Variate: Days_to_Silking
 
Stratum	d.f.	s.e.	cv%
REP	 2	 0.392	 0.6
REP.*Units*	 32	 1.366	 2.2
 
 437  "General Analysis of Variance."
 438  BLOCK REP
 439  TREATMENTS Treatments
 440  COVARIATE "No Covariate"
 441  ANOVA [PRINT=aovtable,information,means,%cv; FACT=32; CONTRASTS=7; PCONTRASTS=7; FPROB=yes;\
 442   PSE=diff,lsd,means; LSDLEVEL=1] Protein_content

Analysis of variance
 
Variate: Protein_content
 
Source of variation	d.f.	s.s.	m.s.	v.r.	F pr.
 
REP stratum	2	 1.2808	 0.6404	 0.67	 
 
REP.*Units* stratum
Treatments	16	 57.0942	 3.5684	 3.72	<.001
Residual	32	 30.6962	 0.9593	 	 
 
Total	50	 89.0712	 	 	 
 
 
Tables of means
 
Variate: Protein_content
 
Grand mean  15.80 
 
	Treatments	 Control	 GT1S1	 GT1S2	 GT2S1	 GT2S2	 GT3S1	 GT3S2
		 16.80	 15.03	 16.05	 14.31	 16.39	 17.22	 17.35
	 
	Treatments	 GT4S1	 GT4S2	 RT1S1	 RT1S2	 RT2S1	 RT2S2	 RT3S1
		 17.54	 15.32	 14.22	 14.80	 16.20	 14.61	 15.39
	 
	Treatments	 RT3S2	 RT4S1	 RT4S2	 	 	 	 
		 16.22	 16.51	 14.70	 	 	 	 
 
 
Standard errors of means
 
Table	Treatments	 
rep.	 3	 
d.f.	 32	 
e.s.e.	 0.565	 
 
 
 
Standard errors of differences of means
 
Table	Treatments	 
rep.	 3	 
d.f.	 32	 
s.e.d.	 0.800	 
 
 
 
Least significant differences of means (1% level)
 
Table	Treatments	 
rep.	 3	 
d.f.	 32	 
l.s.d.	 2.190	 
 
 
 
Stratum standard errors and coefficients of variation
 
Variate: Protein_content
 
Stratum	d.f.	s.e.	cv%
REP	 2	 0.194	 1.2
REP.*Units*	 32	 0.979	 6.2
 
 443  "General Analysis of Variance."
 444  BLOCK REP
 445  TREATMENTS Treatments
 446  COVARIATE "No Covariate"
 447  ANOVA [PRINT=aovtable,information,means,%cv; FACT=32; CONTRASTS=7; PCONTRASTS=7; FPROB=yes;\
 448   PSE=diff,lsd,means; LSDLEVEL=1] Oil_content

Analysis of variance
 
Variate: Oil_content
 
Source of variation	d.f.	s.s.	m.s.	v.r.	F pr.
 
REP stratum	2	 2.3056	 1.1528	 4.88	 
 
REP.*Units* stratum
Treatments	16	 40.4772	 2.5298	 10.71	<.001
Residual	32	 7.5562	 0.2361	 	 
 
Total	50	 50.3390	 	 	 
 
 
Message: the following units have large residuals.
 
REP 2 *units* 7	   -0.842	 s.e.   0.385
REP 3 *units* 7	   1.218	 s.e.   0.385
 
 
Tables of means
 
Variate: Oil_content
 
Grand mean  5.126 
 
	Treatments	 Control	 GT1S1	 GT1S2	 GT2S1	 GT2S2	 GT3S1	 GT3S2
		 4.370	 6.020	 4.770	 5.910	 5.630	 6.180	 4.880
	 
	Treatments	 GT4S1	 GT4S2	 RT1S1	 RT1S2	 RT2S1	 RT2S2	 RT3S1
		 3.760	 4.270	 5.840	 5.910	 4.290	 3.330	 4.700
	 
	Treatments	 RT3S2	 RT4S1	 RT4S2	 	 	 	 
		 5.010	 6.330	 5.940	 	 	 	 
 
 
Standard errors of means
 
Table	Treatments	 
rep.	 3	 
d.f.	 32	 
e.s.e.	 0.2806	 
 
 
 
Standard errors of differences of means
 
Table	Treatments	 
rep.	 3	 
d.f.	 32	 
s.e.d.	 0.3968	 
 
 
 
Least significant differences of means (1% level)
 
Table	Treatments	 
rep.	 3	 
d.f.	 32	 
l.s.d.	 1.0865	 
 
 
 
Stratum standard errors and coefficients of variation
 
Variate: Oil_content
 
Stratum	d.f.	s.e.	cv%
REP	 2	 0.2604	 5.1
REP.*Units*	 32	 0.4859	 9.5
 
 449  "General Analysis of Variance."
 450  BLOCK REP
 451  TREATMENTS Treatments
 452  COVARIATE "No Covariate"
 453  ANOVA [PRINT=aovtable,information,means,%cv; FACT=32; CONTRASTS=7; PCONTRASTS=7; FPROB=yes;\
 454   PSE=diff,lsd,means; LSDLEVEL=1] Starch_content

Analysis of variance
 
Variate: Starch_content
 
Source of variation	d.f.	s.s.	m.s.	v.r.	F pr.
 
REP stratum	2	 1.433	 0.717	 0.71	 
 
REP.*Units* stratum
Treatments	16	 308.226	 19.264	 18.98	<.001
Residual	32	 32.485	 1.015	 	 
 
Total	50	 342.144	 	 	 
 
 
Message: the following units have large residuals.
 
REP 1 *units* 13	   1.72	 s.e.   0.80
REP 3 *units* 13	   -2.24	 s.e.   0.80
 
 
Tables of means
 
Variate: Starch_content
 
Grand mean  67.81 
 
	Treatments	 Control	 GT1S1	 GT1S2	 GT2S1	 GT2S2	 GT3S1	 GT3S2
		 70.12	 66.00	 65.48	 67.84	 68.34	 73.32	 68.70
	 
	Treatments	 GT4S1	 GT4S2	 RT1S1	 RT1S2	 RT2S1	 RT2S2	 RT3S1
		 70.62	 64.52	 65.64	 68.30	 63.20	 67.36	 70.05
	 
	Treatments	 RT3S2	 RT4S1	 RT4S2	 	 	 	 
		 69.60	 65.83	 67.84	 	 	 	 
 
 
Standard errors of means
 
Table	Treatments	 
rep.	 3	 
d.f.	 32	 
e.s.e.	 0.582	 
 
 
 
Standard errors of differences of means
 
Table	Treatments	 
rep.	 3	 
d.f.	 32	 
s.e.d.	 0.823	 
 
 
 
Least significant differences of means (1% level)
 
Table	Treatments	 
rep.	 3	 
d.f.	 32	 
l.s.d.	 2.253	 
 
 
 
Stratum standard errors and coefficients of variation
 
Variate: Starch_content
 
Stratum	d.f.	s.e.	cv%
REP	 2	 0.205	 0.3
REP.*Units*	 32	 1.008	 1.5
 
